# Waveform shaping in photonic time-crystals

**DOI:** 10.1038/s41598-024-53391-8

**Published:** 2024-02-04

**Authors:** Ruey-Bing Hwang

**Affiliations:** Institute of Communications Engineering, College of Electrical and Computer Engineering, National Yang Ming Chiao Tung University, Hsinchu, 300093 Taiwan

**Keywords:** Computational science, Computational methods

## Abstract

This paper reports on the waveform shaped by a finite duration photonic time-crystal with its permittivity and permeability periodically varying in time. A Gaussian-modulated sinusoidal pulse is incident onto this photonic time-crystal to evaluate the backward- and forward-scattering waveforms. An analytical formulation, utilizing a cascade of temporal transfer matrices and the inverse fast Fourier transform, was employed to conduct time-domain waveform computations. Interestingly, the dispersion diagram of the temporal unit cell, which displays a momentum gap characterized by a complex effective angular frequency, plays a crucial role in shaping the incident waveform. Specifically, the presence of momentum gaps in the spectrum of the incident pulse determines the frequencies of the generated oscillation modes.

## Introduction

The research on the propagation of electromagnetic waves in time-varying media can be traced back to Morgenthaler’s 1958 paper^1^. Additionally, the observation of waves exhibiting exponentially increasing amplitudes, known as parametric amplification, within specific wave number ranges in a slab with time-modulated permittivity has been documented^2^. Extensive investigations have been conducted on the scattering characteristics of a plane wave interacting with a slab featuring time-periodic variations in permittivity or permeability^3–5^. Another area of study involves electromagnetic waves propagating in an infinite medium characterized by a traveling-wave modulation of sinusoidal form^6–8^. Additionally, the shape and spectrum of optical pulses can undergo transformations within a linear medium where the refractive index varies with time^9,10^.

In a homogeneous and unbound medium, a sudden change in the permittivity and permeability over time throughout the entire space leads to the generation of reflected and transmitted waves within the same medium, attributed to the temporal interface. The continuity of the electric displacement field (${\textbf{D}}$) and magnetic field (${\textbf{B}}$) was applied at this interface, with constitutive relations ${\textbf{D}}(t)=\varepsilon (t){\textbf{E}}(t)$ and ${\textbf{B}}(t)=\mu (t){\textbf{H}}(t)$, in a linear non-dispersive medium^11^. It is noteworthy that the continuity of $\{{\textbf{E}}, {\textbf{H}}\}$ is unequivocal in dispersive medium such as rapidly growing plasma with ${\textbf{D}}={\textbf{E}}+4\pi \int _{-\infty }^{t} \chi (t,t'){\textbf{E}}(t') dt'$^12^.

Furthermore, the study encompassed an investigation into the reflection and transmission of electromagnetic waves at a temporal boundary, arising from an abrupt change in permittivity and permeability within an unbounded and homogeneous medium^13–15^. The research also delved into the examination of frequency-dependent reflection and transmission coefficients occurring at a temporal boundary within a dispersive medium^16^.

To tailor the propagation properties of electromagnetic waves in materials, the time dimension has been introduced to design dynamic mediums or structures. This approach has been applied in various contexts, including engineering the frequency response of scattered fields from a temporal multilayer structure^17^, designing antireflection coatings^18,19^, synthesizing effective mediums^20,21^, exploring temporal parity-time symmetry^22^, investigating temporal Fabry-Perot cavities^23^, studying photonic time crystals^24,25^, analyzing spatiotemporal dielectric structures^26^, developing quasisonic isolators^27^, creating computational metamaterials^28–30^, and constructing temporal photonic crystals made of dynamic transmission lines^31,32^.

Notably, the photonic temporal crystals examined in this study differ from quantum time crystals, which address the concept of spontaneous time-translation symmetry breaking within quantum mechanics^33,34^.

In this paper, the homogeneous and unbounded medium with abruptly changing in permittivity (refractive index) is considered. Specifically, the temporal variations are periodic with finite cycles (periods). The characteristic solutions, demonstrating the relationship between the effective angular frequency ($\omega _{\text {eff}}$) and incident wave angular frequency, will be calculated. This relationship is crucial for predicting the output waveform when the incident wave spectrum is given. The excitation of modes within the complex $\omega _{\text {eff}}$ (or momentum gap) region significantly influences beam-shaping characteristics.

This paper is structured as follows. In the subsequent section, we will establish both the temporal input-output relation of electric and magnetic fields at a temporal interface, and the temporal transfer matrix representing the wave transition in a temporal slab. These foundational components will enable us to formulate the input-output relation in a photonic time-crystal consisting of finite cycles (temporal slabs). In Section “Results and discussion”, the forward (refracted) and backward (reflected) time-domain waveforms, along with their corresponding spectral responses, will be presented. Additionally, a table illustrating the Root Mean Square error between the outcomes of Finite Difference Time Domain simulation and the method developed in this paper is included. Finally, some concluding remarks on our findings will be provided in Section “Conclusion”.

## Methods

The transfer matrix method has been widely employed for analyzing wave scattering in scenarios that involve cascaded finite thickness uniform dielectric slabs^35–37^. In this section, we apply the temporal analog of the approach^17,19,38–41^ to address the modulation of spatially homogeneous materials with time-varying permittivity $\varepsilon (t)$, permeability $\mu (t)$, or refractive index *n*(*t*). Both $\varepsilon (t)$ and $\mu (t)$ undergo periodic changes with a period of $T_p=1/f_p$, where $f_p$ represents the pumping frequency of this dynamic medium. The homogeneous medium initially possesses parameters $(\mu _1, \varepsilon _1)$ during the first time segment lasting $\tau _1$ seconds, followed by a second time segment with parameters $(\mu _2, \varepsilon _2)$ for a duration of $\tau _2$ seconds.

Firstly, we will employ boundary conditions at a temporal interface to establish an input-output relation for the electric and magnetic fields, expressed in terms of a 2-by-2 matrix denoted as the junction matrix. Additionally, we will derive the transition of electric and magnetic fields within a temporal slab to determine a 2-by-2 transfer matrix. Given that the medium undergoes multiple cycles of changes, one can determine the backward- and forward-scattering waveforms at the steady state of a photonic time-crystal by cascading multiple temporal transfer and junction matrices together.

### Wave scattering at a temporal interface

Consider a monochromatic plane wave traveling in an homogeneous, isotropic, non-dispersive, and unbounded medium; however, the medium is time varying. Its permittivity $\varepsilon$ and permeability $\mu$ can be abruptly switched from $(\mu _1,\varepsilon _1)$ to $(\mu _2,\varepsilon _2)$ at $t=t_o$. In a uniform medium, the electric and magnetic fields can be expressed as $E_x(z,t)=E_1\exp (ik z-i\omega _1 t)$ and $H_y(z,t)=H_1\exp (ik z-i\omega _1 t)$, respectively. Parameters $\omega _1$ is the angular frequency in the initial state and *k* is the wavenumber. The field amplitudes satisfy $Z_1=E_1/H_1$ and $Z_1=\sqrt{\mu _1/\varepsilon _1}$. After the switching event the fields become

1 $$\begin{aligned} E_x(z,t>t_o)=\big [ F e^{-i\omega _2 (t-t_o)}+B e^{i\omega _2(t-t_o)}\big ] E_1 e^{i(k_2 z-\omega _1 t_o)}, \end{aligned}$$

2 $$\begin{aligned} H_y(z,t>t_o)=Y_2\big [ F e^{-i\omega _2 (t-t_o)}-B e^{i\omega _2(t-t_o)}\big ] E_1 e^{i(k_2 z-\omega _1 t_o)}. \end{aligned}$$

Parameters *F* and *B* represent the forward scattering (or transmission) and backward scattering (or reflection) coefficients, respectively. $Z_2=1/Y_2=\sqrt{\mu _2/\varepsilon _2}$ is the wave impedance after the switching event. The temporal boundary conditions, ${\textbf{D}}(t=t_o^-)={\textbf{D}}(t=t_o^+)$ and ${\textbf{B}}(t=t_o^-)={\textbf{B}}(t=t_o^+)$, must be satisfied everywhere in space, leading to $k=k_2$ or $\omega _1\sqrt{\mu _1\varepsilon _1}=\omega _2\sqrt{\mu _2\varepsilon _2}$. This implies momentum conservation across the temporal interface.

The temporal boundary conditions facilitate the formulation of two equations: $\varepsilon _1 E_x(z,t=t_o^-)=\varepsilon _2 E_x(z,t=t_o^+)$ and $\mu _1 H_y(z,t=t_o^-)=\mu _2 H_y(z,t=t_o^+)$, which can be expressed in a matrix-vector form as follows.

3 $$\begin{aligned} \begin{bmatrix} E_x(z,t_o^+) \\ H_y(z,t_o^+) \end{bmatrix}= \begin{bmatrix} \varepsilon _1/\varepsilon _2 &{} 0 \\ 0 &{} \mu _1/\mu _2 \end{bmatrix} \begin{bmatrix} E_x(z,t_o^-) \\ H_y(z,t_o^-) \end{bmatrix} \end{aligned}$$

Equation (3) defines the input-output relation of the electric and magnetic fields at the temporal interface.

### Transfer matrix in a temporal slab

Consider a wave propagating within a temporal slab characterized by a time interval denoted as $\tau$. For instance, the time interval is $t\in (t_o^+,t_o+\tau )$ in Eqs. (1) and (2). The electric and magnetic fields at $t=t_o^+$ can be written as $E_x(z,t=t_o^+)=(F+B) E_1 e^{i(k_2 z-\omega _1 t_o)}$ and $H_y(z,t=t_o^+)=Y_2(F-B) E_1 e^{i(k_2 z-\omega _1 t_o)}$. Therefore, the forward scattering coefficient *F* and backward scattering coefficient *B* can be expressed in terms of $E_x(z,t=t_o^+)$ and $H_y(z,t=t_o^+)$. Substituting the obtained *F* and *B* into Eqs. (1) and  (2) at $t=t_o+\tau$, the electric and magnetic fields evaluated at $t=t_o+\tau$ can be expressed in terms of those at $t=t_o^+$ and written in the temporal transfer matrix given below.

4 $$\begin{aligned} \begin{bmatrix} E_x(z,t_o+\tau ) \\ H_y(z,t_o+\tau ) \end{bmatrix}= \begin{bmatrix} \cos \omega _2\tau &{} -iZ_2\sin \omega _2\tau \\ -iY_2\sin \omega _2\tau &{} \cos \omega _2\tau \end{bmatrix} \begin{bmatrix} E_x(z,t_o^+) \\ H_y(z,t_o^+) \end{bmatrix} \end{aligned}$$

Notably, the result provided by Eq. (4) can be extended to any temporal slab characterized by $(\mu _i,~\varepsilon _i)$, provided that the angular frequency of the wave satisfies $\omega _i\sqrt{\mu _i\varepsilon _i}=k$, where *k* represents the momentum in the initial state.

### A finite cascade of temporal slabs

**Figure 1:**
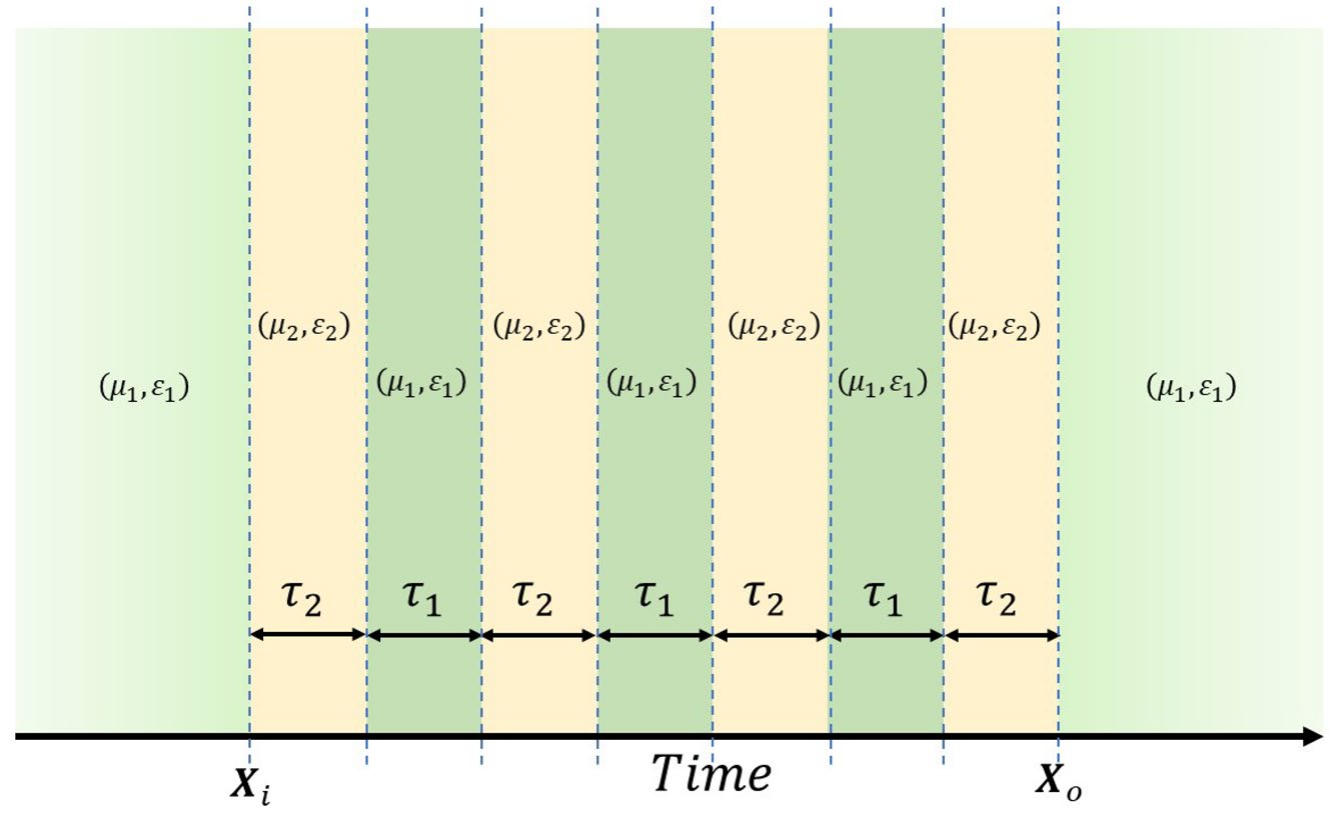
Variation of permeability and permittivity of the temporal photonic crystal against time: it consists of 4 states of $(\mu _2,\varepsilon _2)$ and three states of $(\mu _1,\varepsilon _1)$ in the transition region between $\mathbf {X_o}$ and $\mathbf {X_i}$. The time duration within each temporal slab is denoted as $\tau _1$ and $\tau _2$.

In an unbounded and homogeneous medium, a periodically time-varying scenario emerges where the permeability and permittivity alternate cyclically between $(\mu _1, \varepsilon _1)$ and $(\mu _2, \varepsilon _2)$ with dwell time of $\tau _1$ and $\tau _2$, respectively. The permittivity and permeability (or refractive index) of the medium undergo periodic variations over time, transitioning between these two specified states. For instance, as depicted in Fig. 1, the system comprises four states with parameters $(\mu _2, \varepsilon _2)$ and three states with parameters $(\mu _1, \varepsilon _1)$ within the transition region between ${\textbf{X}}_i$ and ${\textbf{X}}_o$.

The comprehensive input-output relation of $E_x$ and $H_y$ can be derived by sequentially combining those of the constituent components, which encompass the temporal transfer matrix described in Eq. (4) and the junction continuity at the temporal interface outlined in Eq. (3), as elucidated in the preceding sections. This yields the subsequent expression:

5 $$\begin{aligned} {\textbf{X}}_o= {\textbf{C}}_{12}{\textbf{T}}_2{\textbf{C}}_{21} {\textbf{T}}_1{\textbf{C}}_{12}{\textbf{T}}_2{\textbf{C}}_{21} {\textbf{T}}_1{\textbf{C}}_{12}{\textbf{T}}_2{\textbf{C}}_{21} {\textbf{T}}_1{\textbf{C}}_{12}{\textbf{T}}_2{\textbf{C}}_{21}{\textbf{X}}_i \end{aligned}$$

with

6 $$\begin{aligned} {\textbf{C}}_{ij}= \begin{bmatrix} \varepsilon _j/\varepsilon _i &{} 0 \\ 0 &{} \mu _j/\mu _i \end{bmatrix}, \end{aligned}$$

Here, the matrix ${\textbf{C}}_{ij}$ is referred to as the junction matrix at the temporal interface between the *i*-th and *j*-th segments (temporal slab).

7 $$\begin{aligned} {\textbf{T}}_j= \begin{bmatrix} \cos \omega _j T_j &{} -iZ_j\sin \omega _j T_j \\ -iY_j\sin \omega _j T_j &{} \cos \omega _j T_j \end{bmatrix} \end{aligned}$$

where the matrix ${\textbf{T}}_{j}$ is termed as the temporal transfer matrix in the $j$th time segment.

Where ${\textbf{X}}_o=[E_x(t_o+T_e) ~ H_y(t_o+T_e)]^{T}$, ${\textbf{X}}_i=[E_x(t_o^-) ~ H_y(t_o^-)]^{T}$ and $T_e=4(\tau _1+\tau _2)-\tau _1$ represents the total time for cycles. The parameter $Z_j=\sqrt{\mu _j/\varepsilon _j}$ corresponds to the wave impedance in the *j*th temporal slab, where *j*=1 or 2.

Due to momentum conservation, the continuity in *k* between the two states implies that $k=\omega _1\sqrt{\mu _1\varepsilon _1}=\omega _2\sqrt{\mu _2\varepsilon _2}$. This relationship allows us to determine the angular frequency in medium 2 ($\omega _2$) given the angular frequency in medium 1 ($\omega _1$), expressed as $\omega _2=\omega _1\sqrt{\frac{\mu _1\varepsilon _1}{\mu _2\varepsilon _2}}$.

In reality, the aforementioned system comprises three complete cycles (time periods) and one incomplete cycle. However, we can represent it as four cycles and multiply it with ${\textbf{T}}_1^{-1}$, as shown below:

8 $$\begin{aligned} {\textbf{X}}_o={\textbf{T}}_1^{-1} \underbrace{{\textbf{T}}_1{\textbf{C}}_{12}{\textbf{T}}_2{\textbf{C}}_{21}}_{{\textbf{U}}} \underbrace{{\textbf{T}}_1{\textbf{C}}_{12}{\textbf{T}}_2{\textbf{C}}_{21}}_{{\textbf{U}}} \underbrace{{\textbf{T}}_1{\textbf{C}}_{12}{\textbf{T}}_2{\textbf{C}}_{21}}_{{\textbf{U}}} \underbrace{{\textbf{T}}_1{\textbf{C}}_{12}{\textbf{T}}_2{\textbf{C}}_{21}}_{{\textbf{U}}} {\textbf{X}}_i \end{aligned}$$

with

9 $$\begin{aligned} {\textbf{U}}={\textbf{T}}_1{\textbf{C}}_{12}{\textbf{T}}_2{\textbf{C}}_{21} \end{aligned}$$

In this expression, ${\textbf{U}}$ represents the temporal transfer matrix of the unit cell, which comprises two junction matrices and two temporal transfer matrices.

Following this principle, we can extend the approach to a scenario with $N_c$ incomplete cycles, comprising $N_c$ temporal slab 2 and $N_c-1$ temporal slab 1. As a result, the input-output relation of the column vector consisting of $E_x$ and $H_y$ can be formulated as follows:

10 $$\begin{aligned} {\textbf{X}}_o={\textbf{T}}_1^{-1}{\textbf{U}}^{N_c}{\textbf{X}}_i \end{aligned}$$

Based on the theory of eigendecomposition, matrix ${\textbf{U}}$ can be factorized as ${\textbf{U}}={\textbf{P}}{\textbf{D}}{\textbf{P}}^{-1}$.

Where ${\textbf{P}}$ is a matrix whose columns are the eigenvectors of ${\textbf{U}}$, and ${\textbf{D}}$ is a diagonal matrix whose entries of $\lambda _1$ and $\lambda _2$ are the corresponding eigenvalues of ${\textbf{U}}$. Eigendecomposition allows us to simplify and analyze the properties of ${\textbf{U}}$ in terms of its eigenvectors and eigenvalues, making it easier to understand the system’s behavior.

Therefore, Eq. (10) can be rewritten as follows.

11 $$\begin{aligned} {\textbf{X}}_o={\textbf{T}}_1^{-1}{\textbf{P}}{\textbf{D}}^{N_c}{\textbf{P}}^{-1}{\textbf{X}}_i, \end{aligned}$$

with

12 $$\begin{aligned} {\textbf{D}}^{N_c}= \begin{bmatrix} \lambda _1^{N_c} &{} 0 \\ 0 &{} \lambda _2^{N_c} \end{bmatrix}. \end{aligned}$$

This expression substantially reduces the computational complexity involved in calculating the system’s transfer matrix in Eq. (5).

The $E_x$ and $H_y$ in the steady state of the crystal end with parameters $(\mu _1,\varepsilon _1)$ are expressed as follows:

13 $$\begin{aligned} E_x(z,t)=\big [F e^{-i\omega _1 (t-t_o-T_e)}+B e^{+i\omega _1 (t-t_o-T_e)}\big ]e^{ikz}, \end{aligned}$$

and

14 $$\begin{aligned} H_y(z,t)=Y_1 \left[ F e^{-i\omega _1 (t-t_o-T_e)}-B e^{+i\omega _1 (t-t_o-T_e)} \right] e^{ikz} \end{aligned}$$

where $t\ge t_o+T_e$ and $T_e=N_c(\tau _1+\tau _2)-\tau _1$ is the temporal length of the time-crystal.

If we define that

15 $$\begin{aligned} {\textbf{T}}_1^{-1}{\textbf{P}}{\textbf{D}}^{N_c}{\textbf{P}}^{-1}:= \begin{bmatrix} t_{11} &{} t_{12} \\ t_{21} &{} t_{22} \end{bmatrix} \end{aligned}$$

By substituting Eqs. (13), (14) and (15) into Eq. (11), one can determine the forward- and backward-wave amplitudes, shown below.

16 $$\begin{aligned} F=\frac{1}{2} (t_{11}+t_{12} Y_1+ t_{21} Z_1+t_{22})E_1 e^{-i\omega _1 t_o}, \end{aligned}$$

and

17 $$\begin{aligned} B=\frac{1}{2} (t_{11}+t_{12} Y_1- t_{21} Z_1-t_{22})E_1 e^{-i\omega _1 t_o}. \end{aligned}$$

Given that the incident wave can consist of a continuous spectrum, the voltage $E_1$ becomes a function of the incident wave’s angular frequency, denoted as $E_1(\omega _1)$. The system’s response in terms of the forward and backward wave amplitudes will be linked to the spectral function of the incident wave. This perspective enables us to understand the time-domain waveforms of the forward and backward waves under an arbitrary incident waveform, rather than solely focusing on realizing the impulse response of the system.

In this research, a Gaussian-modulated sinusoidal pulse with the spectral function given below is employed as the excitation source (or incident waveform).

18 $$\begin{aligned} E_1(\omega _1)=\frac{\sigma \sqrt{2\pi }}{2i}\exp (-i\omega _1\gamma ) \bigg [ \exp (-\frac{\sigma ^2 (\omega _1-\omega _c)^2}{2})-\bigg [ \exp (-\frac{\sigma ^2 (\omega _1+\omega _c)^2}{2})\bigg ]. \end{aligned}$$

Its corresponding time-domain waveform is

19 $$\begin{aligned} E_1(t)=\exp \bigg [ -\frac{(t-\gamma )^2}{2\sigma ^2}\bigg ]\sin \omega _c(t-\gamma ). \end{aligned}$$

where $\sigma$, $\omega _c$ and $\gamma$ are the pulse width, carrier frequency and offset time of the modulated Gaussian pulse, respectively. It is important to note that a larger bandwidth results in a narrower time pulse width.

Specifically, the forward and backward scattering waveforms can be efficiently computed through the spectral functions of *F* and *B* using the commonly used (IFFT) inverse fast Fourier transform algorithm.

### Complex $\omega _{\text {eff}}$ inside the momentum gap

As mentioned previously, the transfer matrix of the unit cell ${\textbf{U}}$ has two eigenvalues denoted as $\lambda _1$ and $\lambda _2$. These eigenvalues satisfy the following two equations:

20 $$\begin{aligned} \lambda _1+\lambda _2=\text {trace}({\textbf{U}}), \end{aligned}$$

21 $$\begin{aligned} \lambda _1\lambda _2= \det ({\textbf{U}}) =\det ({\textbf{T}}_1) \det ({\textbf{C}}_{12})\det ({\textbf{T}}_2)\det ({\textbf{C}}_{21}) = 1. \end{aligned}$$

where the symbol “trace” represents the trace of a matrix, which is the sum of its diagonal elements. Furthermore, it is known that $\det ({\textbf{C}}{12})\det ({\textbf{C}}{21})=1$, $\det ({\textbf{T}}_1)=1$, and $\det ({\textbf{T}}_2)=1$.

Since $\lambda _1\lambda _2=1$, it allows us to set the eigenvalues ($\lambda$) equal to $\exp (\pm i\omega _{\text {eff}} T_p)$, where $\omega _{\text {eff}}$ represents the effective angular frequency of the unit cell and $T_p=\tau _1+\tau _2$ is the cycle time (time period), and $f_p=1/T_p$ can be interpreted as the pumping frequency of the photonic time crystal (dynamic medium). Now, Eq. (20) can be explicitly written as follows.

22 $$\begin{aligned} \cos (\omega _{\text {eff}} T_p)=\cos \omega _1 \tau _1\cos \omega _2 \tau _2-Q\sin \omega _1 \tau _1\sin \omega _2 \tau _2 \end{aligned}$$

with $$Q=\frac{1}{2}(Z_1/Z_2+Z_2/Z_1)$$.

From Eq. (22), we know that $\omega _{\text {eff}}$ becomes a complex number once the absolute value of the right-hand side term is greater than one. This phenomenon is known as the momentum gap, as reported in the literature. Within the momentum gap, the effective angular frequency is a complex number, and the two eigenvalues are $\exp [\pm i (\omega _r+i\omega _i) T_p]$, where $\omega _r$ and $\omega _i$ are the real and imaginary parts of $\omega _{\text {eff}}$. Due to the relationship $\lambda _1\lambda _2=1$, when one eigenvalue has an amplitude less than one and exhibits exponential decay, the other one has an amplitude greater than one and demonstrates exponential growth.

Notably, the input-output relation in Eq. (11) is related to $\lambda _1^{N_c}$ and $\lambda _2^{N_c}$. These two parameters play an important role in amplitude amplification, known as parametric amplification^42,43^, particularly for the eigenvalue associated with exponential growth. This increase in energy is provided by the pumping energy of the dynamic medium. Accordingly, an increase in $N_c$ enhances the output signal strength, as will be observed in the numerical results.

## Results and discussion

In this section, we explore a photonic time crystal with a periodically changing refractive index over time. This medium exhibits two temporal states of refractive index: $n_1=1.55$ with a dwell time $\tau _1$ and $n_2=1.79$ with a dwell time $\tau _2$. Here, we set $\tau _1 = \tau _2$, resulting in a duty cycle of 50 percent. It’s worth noting that in all the simulation results, time is normalized to $T_p$, and angular frequency is normalized to $\omega _p$.

The incident wave is a Gaussian-modulated sinusoidal pulse with a carrier frequency $\omega _c$ and a bandwidth ranging from $\omega _c-d\omega$ to $\omega _c+d\omega$. The values of $\omega _c$ and $d\omega$ are separately provided in various examples.

Prior to undertaking the detailed waveform analysis, we initially computed the characteristic solution, denoted as $\omega _{\text {eff}}$, from the transfer matrix of the unit cell, as defined in Eq. (22). This preliminary step proved crucial in comprehending the system’s behavior, given that the unit cell transfer matrix functions as the fundamental building block of the entire system.

**Figure 2:**
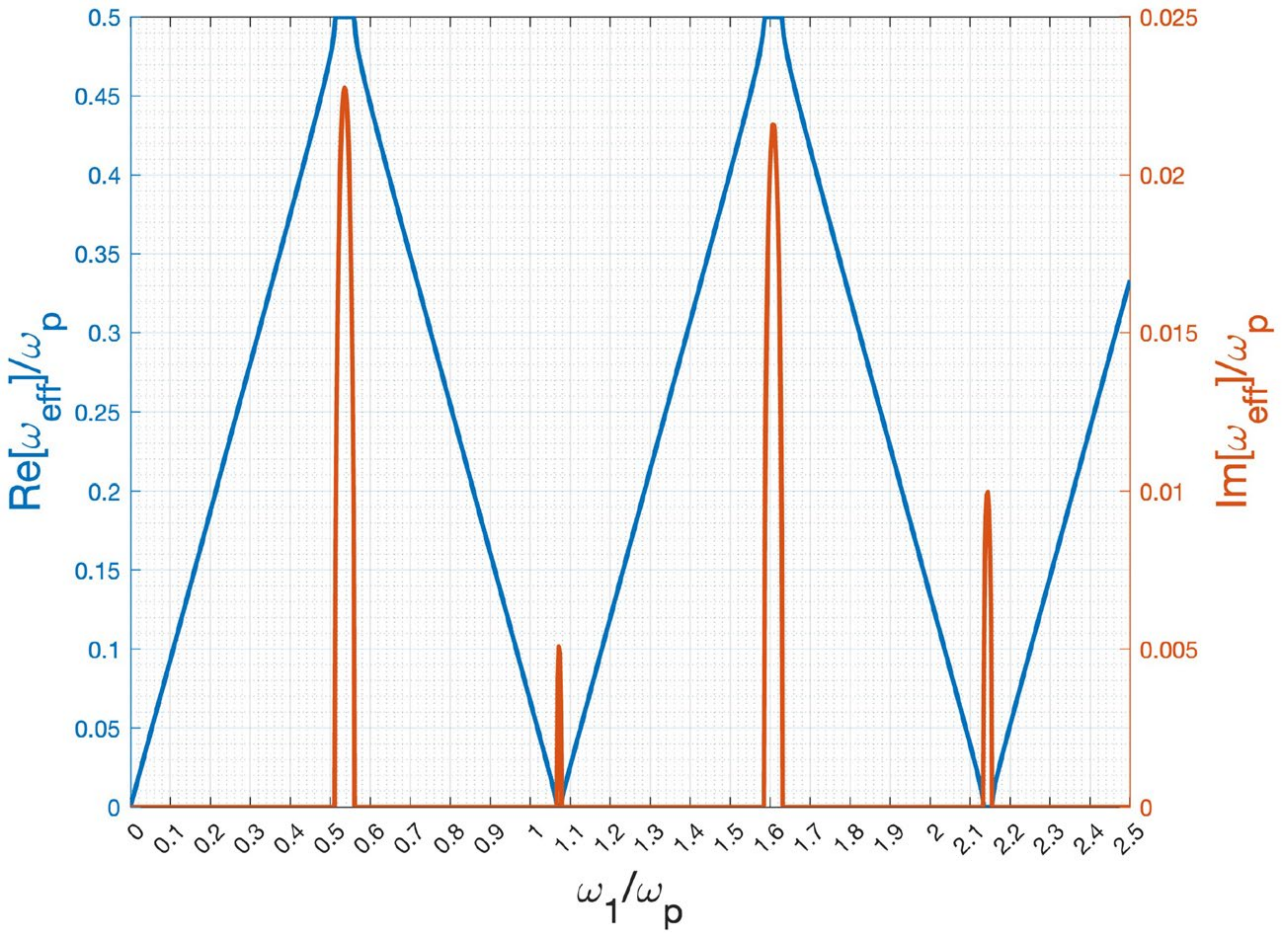
Relationship between normalized effective angular frequency ($\omega _{\text {eff}}/\omega _p$) against the incident wave angular frequency of $\omega _1$ normalized to $\omega _p$: the refractive indices of the two state respectively are $n_1=1.55$, $n_2=1.79$; the time duration of the two states are $\tau _1=\tau _2$. Parameter $\omega _p=2\pi /(\tau _1+\tau _2)$ is the pumping angular frequency of the dynamic medium.

Figure 2 depicts the correlation between the effective angular frequency ($\omega _{\text {eff}}$) and $\omega _1$ (incident wave angular frequency). The blue and red curves represent the real and imaginary components of $\omega _{\text {eff}}/\omega _p$, respectively. Distinct bump-like shapes are clearly observable in the curves, particularly in the momentum gap regions. This phenomenon bears similarity to the behavior exhibited by the imaginary part of the propagation constant within the bandgap of a spatial photonic crystal.

The physical interpretation of $\omega _{\text {eff}}$ lies in its role as the effective or average angular frequency of the wave propagating in the temporal unit cell, analogous to the concept of an effective propagation constant in spatial crystals. While the majority of $\omega _{\text {eff}}$ values are real, there are specific regions where they become complex. This suggests the coexistence of both exponential growth and decay of eigenvalues ($\lambda$). Notably, the exponential growth component significantly contributes to the amplification of an input signal, a point that will be further elucidated in subsequent discussions.

**Figure 3:**
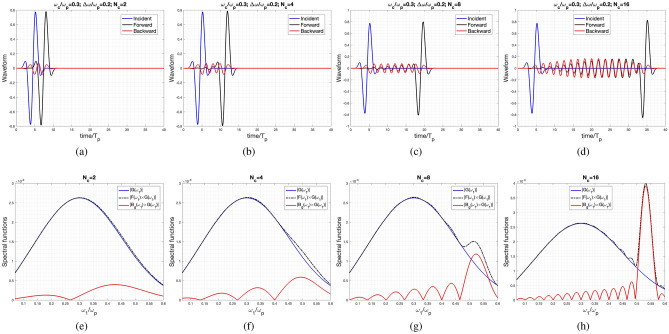
Incident, reflected and transmitted waveform for the case of $\omega _c/\omega _p=0.3$, $d\omega /\omega _p=0.2$ for various cycles: (**a**) $N_c=2$, (**b**) $N_c=4$, (**c**) $N_c=8$, and (**d**) $N_c=16$. Spectral functions of the Incident, reflected and transmitted waves corresponding to the previous waveform for various cycles; where $G(\omega _1)$ is the spectral function of the incident wave. Each sub-figure has the number of cycles of: (**e**) $N_c=2$, (**f**) $N_c=4$, (**g**) $N_c=8$, and (**h**) $N_c=16$.

Figure 3 presents the incident, reflected, and transmitted waveforms plotted against time, normalized to $T_p$. The chosen time range ensures a clear observation of the complete waveform. The center frequency is $\omega _c/\omega _P=0.3$, where $\omega _{\text {eff}}$ is a real number (refer to Fig. 2). However, the signal bandwidth ($d\omega /\omega _p=0.2$) covers the first momentum gap.

In Fig. 3a, it is evident that the forward waveform closely resembles the incident wave, while the backward wave experiences a significant reduction in amplitude. As the number of cycles ($N_c$) increases (from Fig. 3b–d), the forward wave continues to closely mimic the incident wave. However, its oscillating tail becomes more pronounced and extends further with the increase in $N_c$.

Figures from Fig. 3e–h depict the spectral functions of incident, backward, and forward waves corresponding to each case in Figures from Fig. 3a–d. Notably, the spectral function of the incident wave is denoted as $G(\omega _1)$, and both the backward and forward wave spectral functions are multiplied by $G(\omega _1)$.

In Fig. 3e, it is observed that the forward-wave spectral function closely mirrors that of the incident wave, explaining the similarity in their waveforms shown in Fig. 3a. As $N_c$ increases, a noticeable discrepancy between the incident- and forward-wave spectral functions emerges, particularly around the first momentum gap with complex $\omega _{\text {eff}}$. This difference arises due to the excitation of the exponential growth mode, allowing the presence of another Gaussian-modulated sinusoidal pulse with a wider pulse width (due to a narrower bandwidth spectrum in the first momentum gap), as depicted in Fig. 3c and d. Moreover, in Fig. 3h, a narrow-band Gaussian-modulated pulse centered around $\omega _c/\omega _p=0.55$ is superimposed onto the incident-wave spectral function. This corresponds to the wide Gaussian-modulated pulse accompanying the incident pulse, as illustrated in Fig. 3d.

On the other hand, the reflected wave spectral function is dominated by the complex $\omega _{\text {eff}}$ mode, resulting in the appearance of only the wide pulse-width Gaussian-modulated pulse.

**Figure 4:**
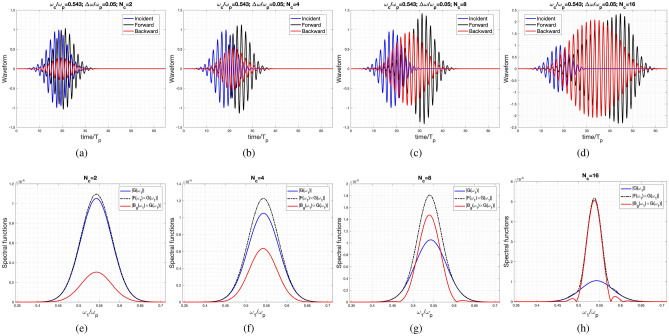
Incident, reflected and transmitted waveform for the case of $\omega _c/\omega _p=0.543$, $d\omega /\omega _p=0.05$ for various cycles: (**a**) $N_c=2$, (**b**) $N_c=4$, (**c**) $N_c=8$, and (**d**) $N_c=16$. Spectral functions of the Incident, reflected and transmitted waves corresponding to the previous waveform for various cycles; where $G(\omega _1)$ is the spectral function of the incident wave. Each sub-figure has the number of cycles of: (**e**) $N_c=2$, (**f**) $N_c=4$, (**g**) $N_c=8$, and (**h**) $N_c=16$.

In the following example, the central frequency of the incident wave is set to $\omega _c/\omega _p=0.543$, strategically positioned at the center of the first momentum gap. This configuration is characterized by a narrow bandwidth of $d\omega /\omega _p=0.05$. From Fig. 4a, it can be observed that the difference between the incident and forward waveforms appears to be insignificant, except for a slight amplitude amplification. The amplitude amplification for the forward wave is evident while the shape of the waveform remains, as depicted in Fig. 4b with $N_c=4$.

In contrast, the two cases with $N_c=8$ and $N_c=16$, illustrated in Fig. 4c and d, reveal a substantial discrepancy between the incident and forward/backward waveforms. In these instances, the carrier frequency remains constant, while the pulse width broadens for both forward and backward waveforms. This observation is supported by their corresponding spectral functions depicted in Fig. 4g and h.

Specifically, in Fig. 4h, an additional narrower Gaussian pulse, originating from the first momentum gap, overlays the incident pulse, aligning their central frequencies. Therefore, it becomes apparent that the pulse width is predominantly determined by the narrow-band component, while maintaining the same central frequency. Additionally, the backward wave exhibits a wider pulse width, a consequence of the narrow-band spectrum displayed in Fig. 4h.

Crucially, both the forward and backward waves demonstrate nearly identical peak spectral strengths within the momentum gap, akin to the characteristics observed in the case of photonic time-crystals with extended temporal durations, as documented in^24^.

### FDTD simulation setup

In addition to the developed approach based on the temporal transfer matrix method, we also employed the finite difference time domain (FDTD) method to validate the obtained waveforms in Figs. 3 and 4. In the one-dimensional FDTD simulation, the Yee algorithm is employed alongside Perfectly Matched Layer (PML) absorbing boundary conditions.

The grid comprises 6799 points, each with a size of $\Delta z=\lambda /200$, where $\lambda$ denotes the wavelength corresponding to the maximum frequency of interest. To ensure numerical stability, the Courant number ($S_c$) is set to unity, with $S_c=c\Delta t/\Delta z=1$, where *c* represents the speed of light. There are 64 PML cells placed on both sides to terminate the grid. The excitation source is located at $200\Delta z$, and the probes are strategically positioned at $6686\Delta z$ and $114\Delta z$ to record transmission (forward-scattering) and reflection (backward-scattering) waves, respectively.

The permittivity $(\varepsilon )$ of the medium is time-dependent, changing periodically between values $\varepsilon _1$ and $\varepsilon _2$ over a cycle $T_p$, while the nonmagnetic material with $\mu =\mu _o$ is considered. Both the initial and final states are characterized by $\varepsilon =\varepsilon _1$. The simulation spans 15,000 iterations, providing a comprehensive exploration of the dynamics of the electromagnetic field.

Table 1 presents the Root Mean Square (RMS) values for the differences between the waveforms obtained by the FDTD method and those derived from our approach, considering both forward- and backward-scattering waves for various values of $N_c$. Furthermore, the results obtained by both the FDTD method and the approach developed in this paper, along with their respective absolute differences, are plotted and shown in the Supplementary material.

Furthermore, Table 1 highlights a notable discrepancy between the results depicted in Fig. 3 obtained from the two approaches, particularly in the case of the forward-scattering wave. If we consider the transfer matrix approach as a more rigorous method, the observed deviation in the FDTD simulation might be attributed to the challenges associated with wide bandwidth operation. This significant deviation can be attributed to the fact that the segment of the narrow time pulse is being mimicked by the forward wave, copying the wide spectral response of the incident wave.

However, in the case of the backward wave, which only excites the wide pulse (or narrow spectral bandwidth) contributed by the complex $\omega _{\text {eff}}$ within the momentum gap, the error is relatively small compared to that of the forward waves. Additionally, noticeable RMS errors are observed in the cases of $N_c=8$ and $N_c=16$, indicating obvious parametric amplification.

**Table 1:** The table presents the Root Mean Square (RMS) error between outcomes obtained from Finite Difference Time Domain (FDTD) simulations and the approach presented in this paper.

	Fig. 3 (FW)	Fig. 4 (FW)	Fig. 3 (BW)	Fig. 4 (BW)
$N_c=2$	$6.38\times 10^{-3}$	$9.18\times 10^{-4}$	$5.24\times 10^{-4}$	$3.59\times 10^{-4}$
$N_c=4$	$4.88\times 10^{-3}$	$4.14\times 10^{-3}$	$8.34\times 10^{-4}$	$2.79\times 10^{-3}$
$N_c=8$	$8.18\times 10^{-3}$	$9.79\times 10^{-3}$	$6.97\times 10^{-4}$	$9.21\times 10^{-3}$
$N_c=16$	$4.75\times 10^{-3}$	$4.55\times 10^{-2}$	$3.02\times 10^{-3}$	$4.36\times 10^{-2}$

The forward- and backward-scattering waveforms are individually labeled as FW and BW in the table.

**Figure 5:**
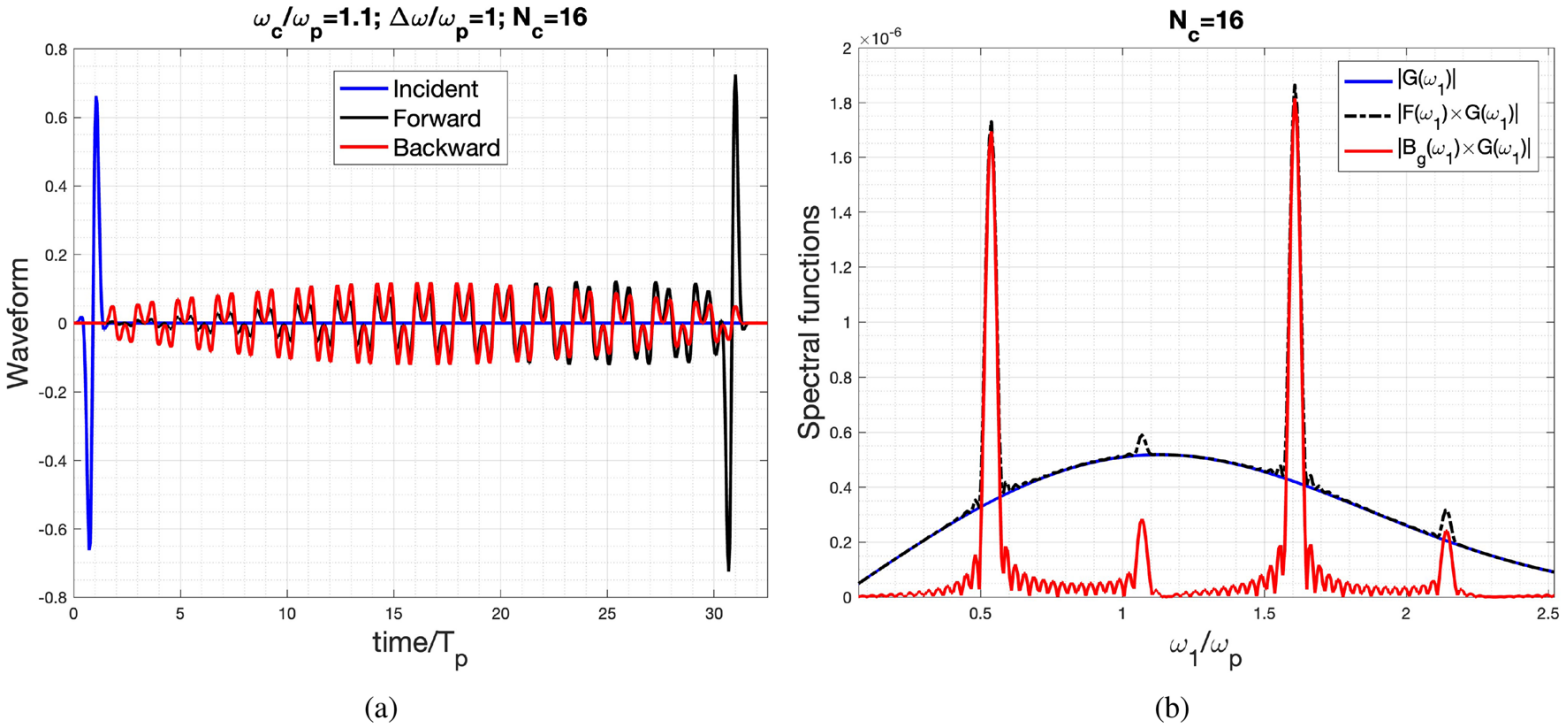
Incident, reflected and transmitted response for the case of $\omega _c/\omega _p=1.1$, $d\omega /\omega _p=1$ for $N_c=16$ (**a**) time-domain waveform and (**b**) spectral function.

The current example involves an ultra-wideband pulse incident into the temporal crystal. Notably, an ultrashort pulse can be employed to fully characterize linear photonic devices^44^. The central frequency is set at $\omega _c/\omega _p=1.1$ with $d\omega /\omega _p=1.0$. Figure 5a reveals that the initial segment of the forward wave replicates the incident pulse, albeit with minor amplitude deviations. This observation is supported by its spectral functions, as depicted in Fig. 5b. Evidently, the forward-wave spectrum encapsulates the profile of the incident wave.

Notably, the two oscillating modes characterized by larger imaginary parts of $\omega _{\text {eff}}$ (as depicted in Fig. 2) exhibit more pronounced spectral amplitudes within the forward and backward waves. This phenomenon becomes particularly evident in the extended tail featuring two distinct frequencies, which is clearly observable in Fig. 5a.

Furthermore, the RMS of the difference between the results obtained by the FDTD method and the approach employed in this paper has been calculated. The RMS of the differences is $1.76\times 10^{-2}$ for the forward propagating waveform and $4.24\times 10^{-3}$ for the backward propagating wave, respectively.

Interestingly, in the momentum gaps with a significant imaginary part of $\omega _{\text {eff}}$, such as the first and third ones, the forward and backward waves almost have equal amplitudes. This is particularly evident in the cases with $N_c=16$, as clearly observed in Figs. 3h, 4h, and 5b.

**Figure 6:**
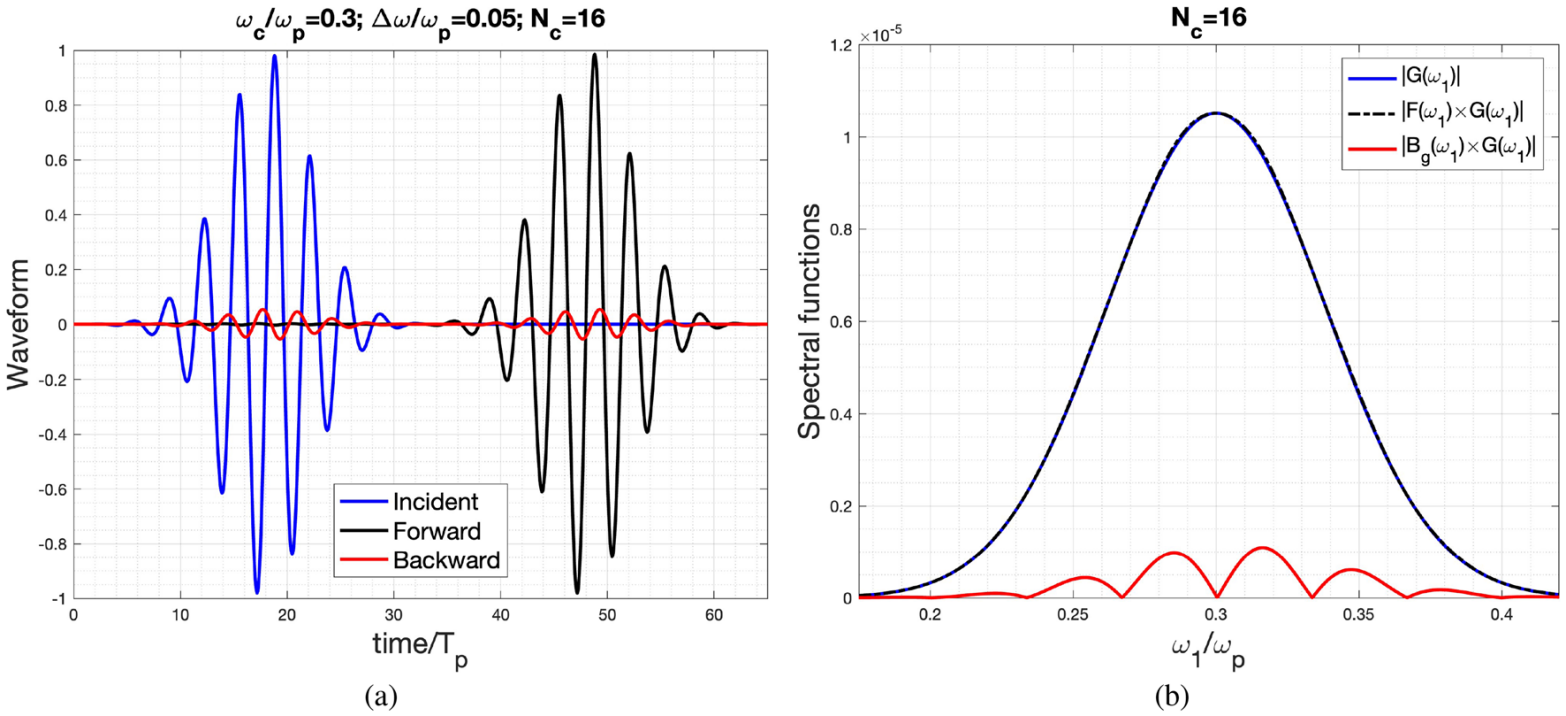
Waveform and Spectral functions of the Incident, reflected and transmitted waves for the case of $\omega _c/\omega _p=0.3$, $d\omega /\omega _p=0.05$ for $N_c=16$. Subfigures (**a**) is the time-domain waveforms and (**b**) is the corresponding spectral functions.

In the previously mentioned instances, the spectrum of the incident wave spans the momentum gaps. However, in the last example, where the central frequency and half-bandwidth are defined as $\omega _c/\omega _p=0.3$ and $d\omega /\omega _p=0.05$ respectively, it fails to encompass any momentum gaps. Notably, the forward wave replicates the incident waveform, as demonstrated in Fig. 6a. Additionally, the spectral functions of the incident and forward waves coincide, as depicted in Fig. 6b. The RMS value of the difference between the results of FDTD and this approach is $7.92\times 10^{-3}$ (for the forward-scattering wave) and $2.12\times 10^{-4}$ (for the backward-scattering wave), respectively.

## Conclusion

In this study, we have developed a comprehensive mathematical framework and a rigorous formulation to analyze the waveforms of both forward and backward scattering within a photonic time-crystal with finite cycles (periods). The approach involves utilizing a cascade of temporal slabs based on the commonly used transfer matrix method to systematically establish the temporal input-output relationship for electric and magnetic field spectral functions in an environment that encompasses multiple temporal interfaces. Moreover, by incorporating the inverse fast Fourier transform, an efficient extraction of the corresponding time-domain waveforms can be achieved.

It is noteworthy that this methodology distinguishes itself from the finite-difference time-domain (FDTD) method, offering a unique avenue for gaining deeper physical insights into the intricate dynamics of wave propagation through photonic time-crystals. Furthermore, the FDTD simulation has been employed to validate the analytical results.

The observations reveal that waveform shaping becomes evident when the spectrum of an incident wave covers the momentum gaps, with particularly distinct effects observed in instances characterized by a high number of cycles ($N_c$). Moreover, remarkable parametric amplification occurs when the incident spectrum is situated within the momentum gaps and spans extended cycles. Additionally, the introduction of extra modulated-Gaussian pulses takes place when an incident spectrum encompasses momentum gaps. This particular situation holds significant promise for applications in detecting mediums that display time-periodic changes in permittivity and permeability, especially within the domain of non-destructive testing.

### Supplementary Information

Supplementary Information.

## Data Availability

The data supporting the findings of this paper are available from the corresponding author upon reasonable request.
