# Supplementary material for: Waveform shaping in photonic time-crystals
Source: Sci Rep. 2024 Feb 4;14:2864. doi: 10.1038/s41598-024-53391-8 (PMC10838957; doi:10.1038/s41598-024-53391-8)
Supplement: Supplementary file 1 — Supplementary Information. [file 41598_2024_53391_MOESM1_ESM.pdf]

Waveform Shaping in Photonic Time-Crystals: Supplementary Material

Ruey-Bing Hwang
Institute of Communications Engineering
National Yang Ming Chiao Tung University, Hsichu, Taiwan

Finite Difference Time Domain Simulation

Simulation Configuration

A one-dimensional Finite Difference Time Domain (FDTD) simulation is implemented using the Yee algorithm, incorporating Perfectly Matched Layer (PML) absorbing boundary conditions. Here are the key parameters:

- **Grid Setup**:
  - Total grids: 6799
  - Grid size: $\Delta z = \lambda/200$, where λ is the wavelength corresponding to the maximum frequency.
- **Numerical Stability**
  - Courant number (S_c): $S_c = \frac{c\Delta t}{\Delta z} = 1$, ensuring stability, where c is the light speed.
- **Boundary conditions**
  - Perfect Matched Layer (PML) cells: 64 on both sides to terminate grid
- **Locations of the Excitation and Probes**
  - Excitation source: $200\Delta z$
  - Probes for recording transmission (at $6686\Delta z$) and reflection (at $114\Delta z$) waves
- **Material Characteristics**
  - permittivity (ε)
    * Time-dependent
    * Periodically varies between ε_1 and ε_2 with a cycle of T_p

* Initial and final states have $\varepsilon = \varepsilon_1$

- **Simulation Parameters**
  - Number of iterations: 15000

Numerical Results

The numerical results obtained by the FDTD method and the approach shown in this paper, along with their differences, for the figures in Fig. 3 and Fig. 4 are plotted as follows.

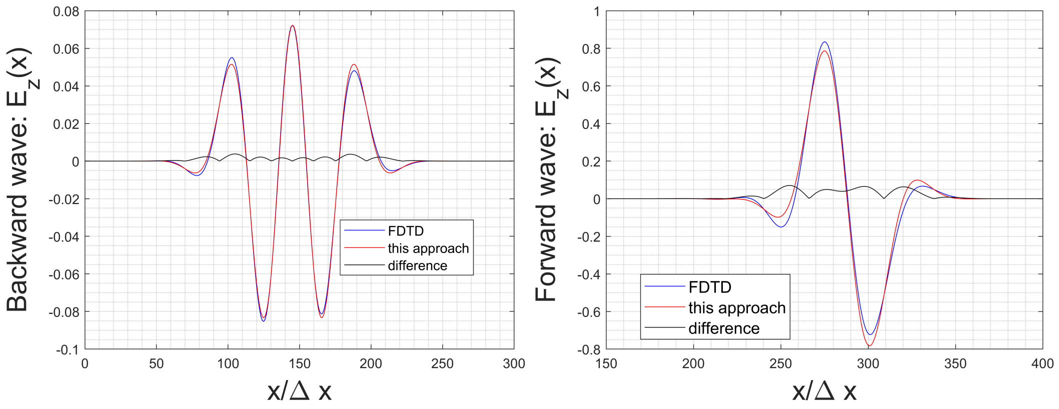

(a) (b)

Figure 1: Waveforms calculated by FDTD and temporal transfer matrix method, together with their absolute differences: corresponding the Fig. 3(a); (a) backward scattering wave and (b) forward scattering wave.

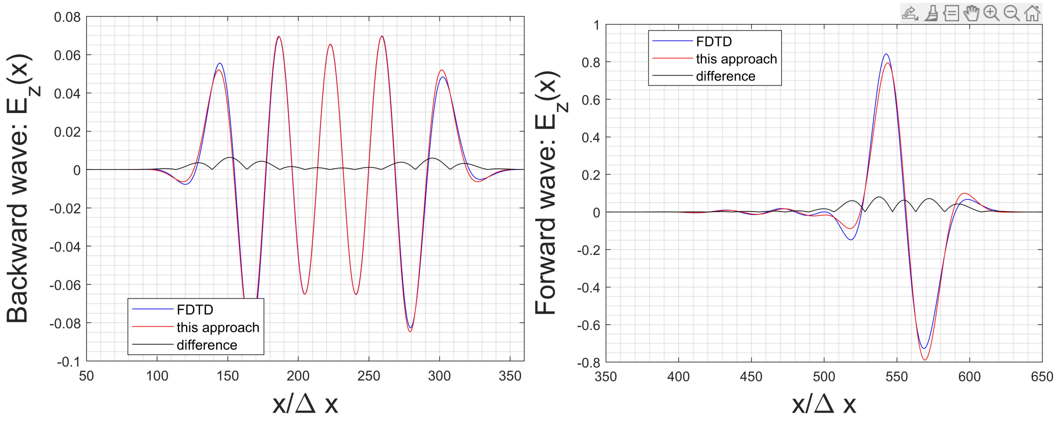

(a) (b)

Figure 2: Waveforms calculated by FDTD and temporal transfer matrix method, together with their absolute differences: corresponding the Fig. 3(b); (a) backward scattering wave and (b) forward scattering wave.

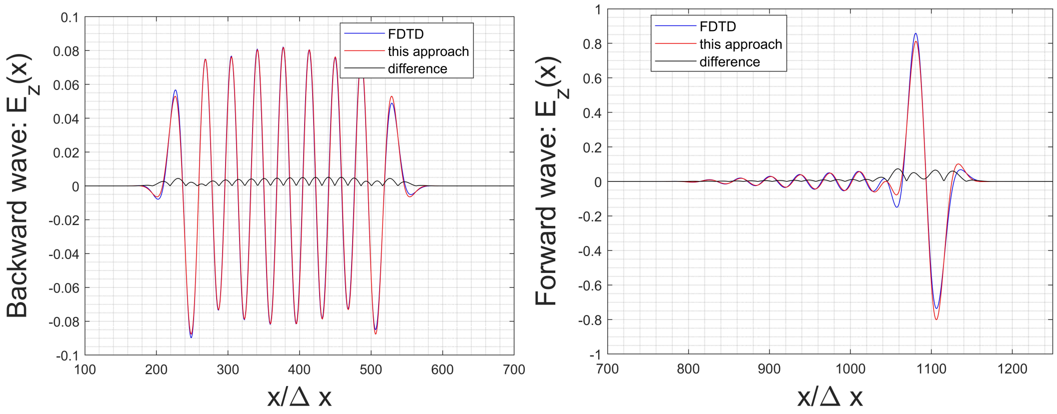

(a) (b)

Figure 3: Waveforms calculated by FDTD and temporal transfer matrix method, together with their absolute differences: corresponding the Fig. 3(c); (a) backward scattering wave and (b) forward scattering wave.

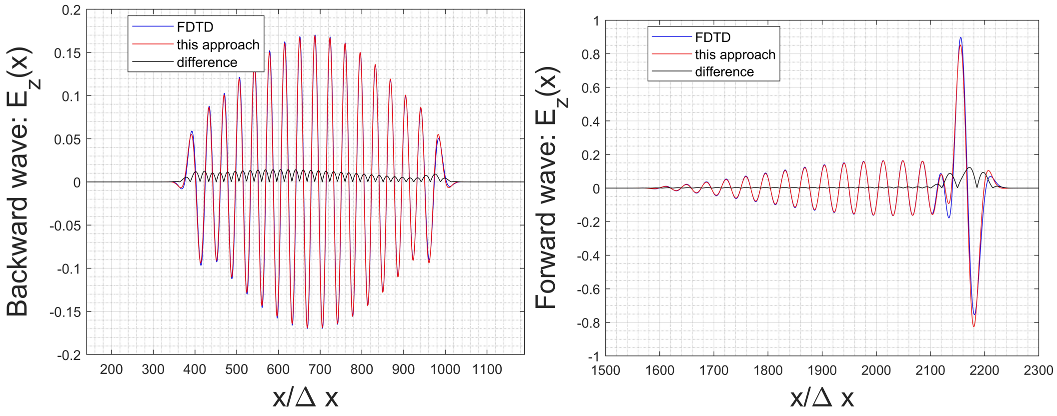

(a) (b)

Figure 4: Waveforms calculated by FDTD and temporal transfer matrix method, together with their absolute differences: corresponding the Fig. 3(d); (a) backward scattering wave and (b) forward scattering wave.

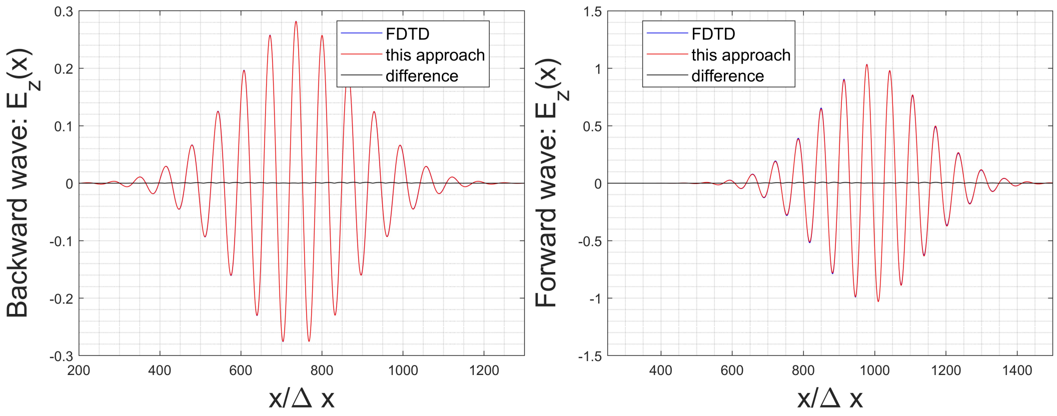

(a) (b)

Figure 5: Waveforms calculated by FDTD and temporal transfer matrix method, together with their absolute differences: corresponding the Fig. 4(a); (a) backward scattering wave and (b) forward scattering wave.

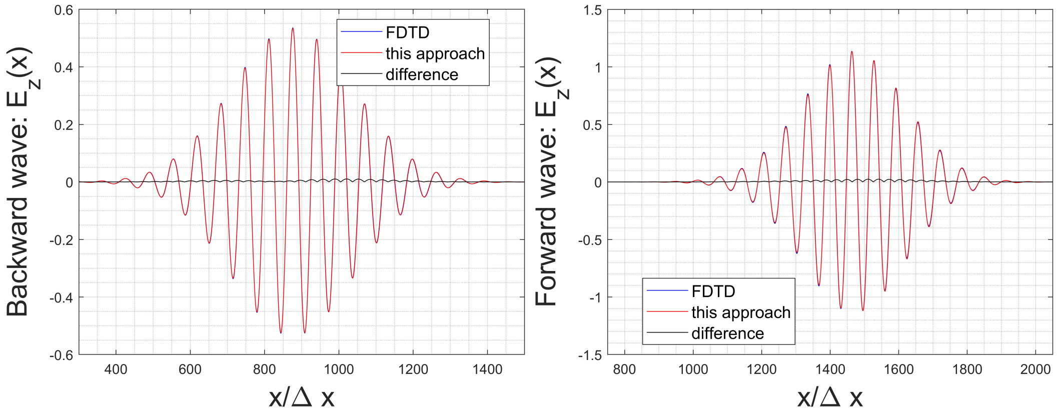

(a) (b)

Figure 6: Waveforms calculated by FDTD and temporal transfer matrix method, together with their absolute differences: corresponding the Fig. 4(b); (a) backward scattering wave and (b) forward scattering wave.

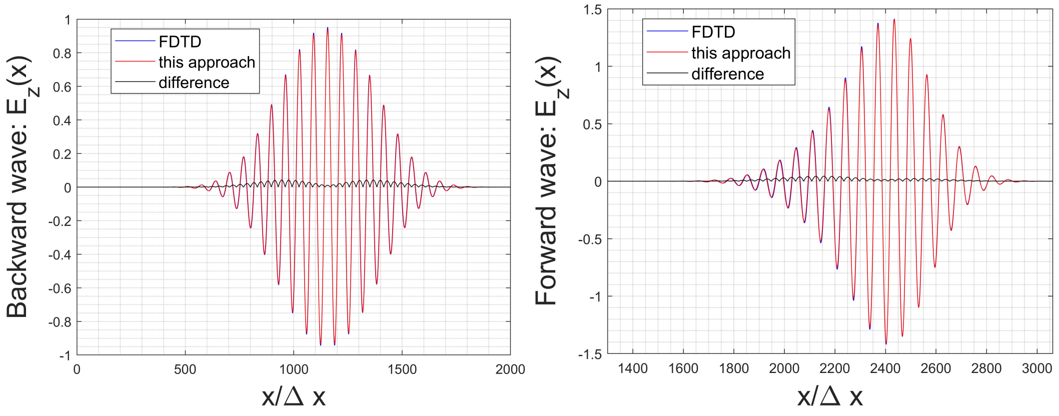

(a) (b)

Figure 7: Waveforms calculated by FDTD and temporal transfer matrix method, together with their absolute differences: corresponding the Fig. 4(c); (a) backward scattering wave and (b) forward scattering wave.

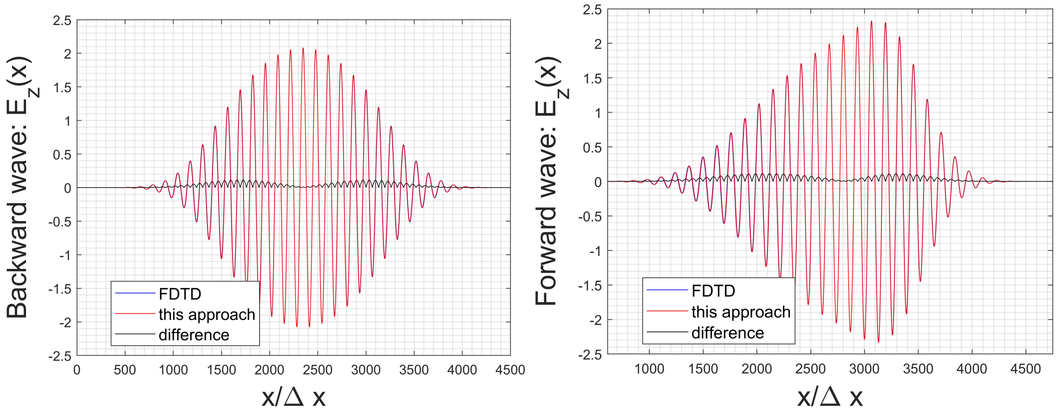

(a) (b)

Figure 8: Waveforms calculated by FDTD and temporal transfer matrix method, together with their absolute differences: corresponding the Fig. 4(d); (a) backward scattering wave and (b) forward scattering wave.
